# The interplay between gray matter and white matter neurodegeneration in subjective cognitive decline

**DOI:** 10.18632/aging.203467

**Published:** 2021-08-25

**Authors:** Nira Cedres, Patricia Diaz-Galvan, Lucio Diaz-Flores, J-Sebastian Muehlboeck, Yaiza Molina, José Barroso, Eric Westman, Daniel Ferreira

**Affiliations:** 1 Division of Clinical Geriatrics, Centre for Alzheimer Research, Department of Neurobiology, Care Sciences, and Society (NVS), Karolinska Institutet (KI), Stockholm, Sweden; 2 Department of Psychology, Sensory Cognitive Interaction Laboratory (SCI-lab), Stockholm University, Stockholm, Sweden; 3 Department of Radiology, Mayo Clinic, Rochester, MN 55905, USA; 4 Hospital Universitario de Canarias, La Laguna, Tenerife, Spain; 5 Faculty of Health Sciences, University Fernando Pessoa Canarias, Las Palmas de Gran Canaria, Spain; 6 Faculty of Psychology, University of La Laguna, La Laguna, Tenerife, Spain; 7 Department of Neuroimaging, Centre for Neuroimaging Sciences, Institute of Psychiatry, Psychology and Neuroscience, King’s College London, London, UK

**Keywords:** subjective cognitive decline, gray matter, white matter, aging, mediation

## Abstract

Aims: To investigate the interplay between gray matter (GM) and white matter (WM) neurodegeneration in subjective cognitive decline (SCD), including thickness across the whole cortical mantle, hippocampal volume, and integrity across the whole WM.

Methods: We included 225 cognitively unimpaired individuals from a community-based cohort. Subjective cognitive complaints were assessed through 9 questions covering amnestic and non-amnestic cognitive domains. In our cohort, 123 individuals endorsed from one to six subjective cognitive complaints (i.e. they fulfilled the diagnostic criteria for SCD), while 102 individuals reported zero complaints. GM neurodegeneration was assessed through measures of cortical thickness across the whole mantle and hippocampal volume. WM neurodegeneration was assessed through measures of mean diffusivity (MD) across the whole WM skeleton. Mediation analysis and multiple linear regression were conducted to investigate the interplay between the measures of GM and WM neurodegeneration.

Results: A higher number of complaints was associated with reduced hippocampal volume, cortical thinning in several frontal and temporal areas and the insula, and higher MD across the WM skeleton, with a tendency to spare the occipital lobe. SCD-related cortical thinning and increased MD were associated with each other and jointly contributed to complaints, but the contribution of cortical thinning to the number of complaints was stronger.

Conclusions: Neurodegeneration processes affecting the GM and WM seem to be associated with each other in SCD and include brain areas other than those typically targeted by Alzheimer’s disease. Our findings suggest that SCD may be a sensitive behavioral marker of heterogeneous brain pathologies in individuals recruited from the community.

## INTRODUCTION

Multiple pathologies can co-exist in cognitively unimpaired individuals, causing neurodegeneration years before the onset of cognitive decline [1]. Increasing research is trying to ascertain whether individuals are able to subjectively detect such neurodegeneration, motivating the emergence of concepts like subjective cognitive decline (SCD), as a risk factor for dementia [2–4]. Several studies showed that SCD may be a harbinger of Alzheimer’s disease (AD) [5–7]. However, community-based studies show that SCD can also be associated with cerebrovascular disease [8–10], age-related tauopathy [11], and emotional factors such as depressive symptomatology [9, 12, 13].

Neurodegeneration can be assessed *in vivo* with magnetic resonance imaging (MRI)*.* Previous studies revealed macrostructural neurodegeneration in the brain gray matter (GM) of SCD individuals, but the analyses were often limited to areas typically affected in AD. These studies consistently found reduced volumes in the hippocampus and entorhinal cortex [14–16], and cortical thinning in medial temporal areas [17–19]. Other studies expanded these analyses to include the entire cortical mantle by investigating AD-like atrophy patterns [20, 21]. However, investigating AD-related brain areas or AD-like atrophy patterns may hinder the possibility to detect neurodegeneration related to non-AD pathologies in SCD. Some studies overcame this limitation by exploring the whole cortex using voxel-based morphometry or vertex-wise analysis in SCD [22–29]. While some authors reported reduced GM volume or thickness in hippocampus, precuneus, cingulum and frontal cortex in SCD individuals compared with healthy controls [22, 28], other authors reported no differences [24, 25], or even increased GM volume in fusiform gyrus and occipital areas in SCD [26, 27]. In addition, previous studies operationalized SCD mostly based on episodic memory complaints and had a strong focus on AD. As a result, little is known about GM neurodegeneration potentially associated with complaints in non-memory cognitive domains.

In addition, several SCD studies investigated neurodegeneration in the white matter (WM) by using diffusion tensor imaging (DTI). The scarce data available suggest neurodegeneration in several WM areas in SCD [22, 23, 30–33]. However, some other DTI studies reported no WM neurodegeneration in SCD [24, 34]. An important question that remains unanswered is how WM and GM neurodegeneration relate to each other in SCD individuals. This question is relevant in order to elucidate the earliest stages of overt neurodegeneration in individuals at risk of dementia. So far, this question has only been investigated in one previous study [23]. Hong et al. (2016) investigated 46 SCD patients, of which 19 had a high risk of progressing to AD and 27 had a low risk of progressing to AD based on age, *APOE* genotype, and cognitive performance. Using DTI, Hong et al. (2016) showed that SCD patients at a high risk of progressing to AD had greater neurodegeneration in frontotemporal WM areas, while no differences were found in cortical thickness.

The overall goal of the current study was to extend the previous research on GM and WM neurodegeneration in SCD. To do that, we (i) investigated cortical thickness across the whole mantle, hippocampal volume, and integrity across the whole WM skeleton, and (ii) studied the interplay between GM and WM neurodegeneration. SCD was operationalized through complaints in several cognitive domains, not only episodic memory, in a community-based cohort of 225 individuals. Since age is a major contributor to GM neurodegeneration [35], WM neurodegeneration [36], and subjective cognitive complaints [9], we also investigated the role of age in this study. Firstly, we used multiple linear regression models and Pearson correlations to analyze the association between age and thickness across the whole cortical mantle, hippocampal volume, and integrity across the whole WM skeleton. Secondly, we conducted mediation analyses to investigate the interplay between GM and WM neurodegeneration, with and without age as a covariate.

## RESULTS

Two hundred and twenty-five cognitively unimpaired participants (mean age 54.7 years, range from 35 to 77 years, 55% female) were included in the current study. The demographics and clinical characteristics of the cohort are summarized in Table 1. A total of 123 participants reported between 1 and 6 complaints (mean (SD)=1.8(0.9)), whereas 102 participants reported 0 subjective cognitive complaints. The mean (SD) of subjective cognitive complaints for the whole sample was 0.9 (1.1). Naming (40%) and memory (28%) were the most frequent subjective cognitive complaints.

**Table 1:** Demographic and clinical characteristics.

	**Mean (SD) / percentage**	**Min-max**
Age	54.64 (10.18)	35 - 77
Sex (% women)	55	-
Education level (% 0/1/2/3/4)^1^	0/3/35/25/37	-
Information (WAIS-III)	16.82 (6.00)	5 - 27
MMSE	28.91 (1.19)	24 - 30
BDRS	0.58 (0.91)	0 – 3.50
FAQ	0.30 (0.67)	0 - 5
Subjective cognitive complaints^2^	0.92 (1.1)	0 - 6
Depressive symptomatology^3^	0 (1)	-1.20 – 3.76
Global MD^4^	7.43 (0.22)	6.78 – 8.21
WMSA volume	2183.42 (1951.94)	471 - 12677

^1^Education Level: illiterate (0); acquired reading and/or writing skills (1); primary level (2); secondary level (3); university level (4).

^2^Subjective cognitive complaints were studied through nine yes/no questions as explained in the methods.

^3^Depressive symptomatology was estimated by transforming BDI and GDS scores into z scores and then combined them into one single variable.

^4^MD values were multiplied by 10000.

WAIS, Wechsler Adult Intelligence Scale; MMSE, Mini-Mental State Examination; BDRS, Blessed Dementia Rating Scale; FAQ, Functional Activity Questionnaire; BDI, Beck Depression Inventory; GDS, Geriatric Depression Scale; WMSA, White Matter Signal Abnormalities; MD, Mean Diffusivity.

### The association between GM neurodegeneration and subjective cognitive complaints

**Figure 1:**
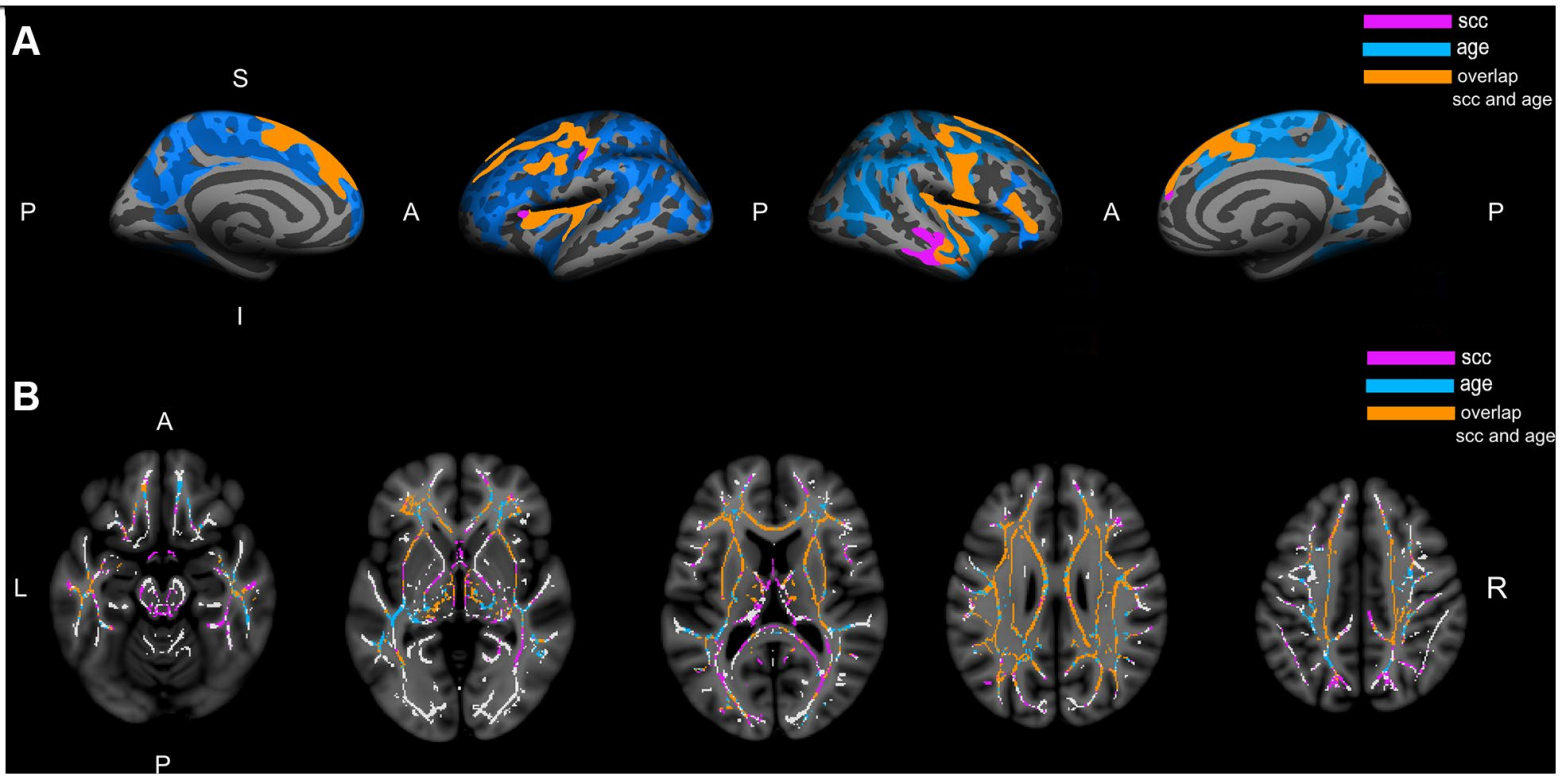
**Association of subjective cognitive complaints and age with cortical thickness and white matter integrity.** (**A**) Represents the cortical thinning exclusively associated with subjective cognitive complaints (pink), the cortical thinning exclusively associated with age (blue), and the cortical thinning associated with both complaints and age (orange). (**B**) Represents the increase in mean diffusivity exclusively associated with subjective cognitive complaints (pink), the increase in mean diffusivity exclusively associated with age (blue), and the increase in mean diffusivity associated with both complaints and age (orange); The white matter skeleton is represented in white color. All the represented clusters are statistically significant at p>0.01 after correction for multiple testing; A: anterior; P: posterior; S: superior; I: inferior; L: left; R: right; SCC: subjective cognitive complaints.

The vertex-wise analysis showed that a higher number of subjective cognitive complaints was significantly associated with reduced cortical thickness in 11 clusters including lateral and medial frontal areas and the insula of both hemispheres, and lateral temporal areas in the right hemisphere (Table 2 and Figure 1A). The vertex-wise analysis fitted for age showed that older age was significantly associated with reduced cortical thickness across the whole cortex with a tendency to spare the occipital lobe (Figure 1A). Figure 1A shows the overlap between the cortical maps related to the number of subjective cognitive complaints and age. As it can be seen, most of the cortical areas related to the number of subjective cognitive complaints were also related to age (Figure 1A).

**Table 2:** The association between subjective cognitive complaints and cortical thickness.

**Cluster #**	**Max^1^**	**Brain area^2^**	**Size (mm^2^)**	**MNIX**	**MNIY**	**MNIZ**	**p-value**
*Left hemisphere*							
1	-6.493	Superior frontal	2287.9	-6.9	39.4	38.3	<0.001
2	-4.613	Precentral	2077.6	-36.0	-11.7	50.1	<0.001
3	-5.811	Pars opercularis	1209.5	-35.8	14.2	9.9	<0.001
4	-3.536	Caudal middle frontal	605.1	-37.9	0.9	30.8	0.003
5	-4.634	Paracentral	413.8	-18.0	-32.9	43.2	0.032
*Right hemisphere*							
1	-5.400	Precentral	2537.1	52.5	-2.9	34.4	<0.001
2	-4.052	Superior frontal	1152.3	12.8	4.5	40.1	<0.001
3	-4.339	Superior temporal	1094.8	52.1	-11.0	-8.8	<0.001
4	-3.984	Caudal middle frontal	728.8	26.9	-0.5	41.9	<0.001
5	-4.089	Superior frontal	722.1	23.0	4.6	57.5	<0.001
6	-4.469	Pars triangularis	634.9	45.6	35.4	-5.8	0.002

^1^The Max indicates the maximum log10(p) value across the vertices in the cluster.

^2^Location of the peak voxel as per the Desikan atlas in FreeSurfer. The MNI coordinates indicate the location of the peak vertex. P-values are cluster-wise.

The Pearson correlation for the association between complaints and hippocampal volume showed that the number of subjective cognitive complaints was negatively associated with hippocampal volume (Table 3). The Pearson correlation for the association between age and hippocampal volume showed that age was negatively associated with hippocampal volume (Table 3).

**Table 3:** Correlation matrix for the average MD in significant clusters, average cortical thickness in significant clusters, age, and complaints.

	**SCC**	**Age**	**Average MD**
**Age**	0.37***	-	-
**Average MD**	0.36***	0.55***	-
**Average cortical thickness**	-0.49***	-0.60***	-0.47***
**Hippocampal volume**	-0.14*	-0.29***	-0.40***

SCC, subjective cognitive complaints; MD, mean diffusivity; ***p<0.001; *p<0.05.

### The association between WM neurodegeneration and subjective cognitive complaints

The voxel-based tract-based spatial statistics (TBSS) analysis showed that a higher number of subjective cognitive complaints was significantly associated with a higher mean diffusivity (MD) in one large cluster involving most of the WM skeleton, with a tendency to spare the occipital lobe (Figure 1B). The voxel-based TBSS analysis fitted for age showed that an older age was significantly associated with higher MD in one large cluster involving most of the WM skeleton, with a tendency to spare the occipital and parietal lobes, as well as to spare tracts going through the internal capsule (Figure 1B). The map of WM tracts related to subjective cognitive complaints showed that a higher MD in the internal capsule and posterior white matter tracts (i.e., splenium of the corpus callosum, posterior portion of the superior longitudinal fasciculus, posterior thalamic radiation, and forceps major) was associated with a higher number of complaints. These areas were not significant in the map of WM tracts related to age (Figure 1B).

### The interplay between GM neurodegeneration and WM neurodegeneration related to subjective cognitive complaints

The average cortical thickness, hippocampal volume, and average MD of the statistically significant clusters reported above were used as the input data for mediation analysis.

**Figure 2:**
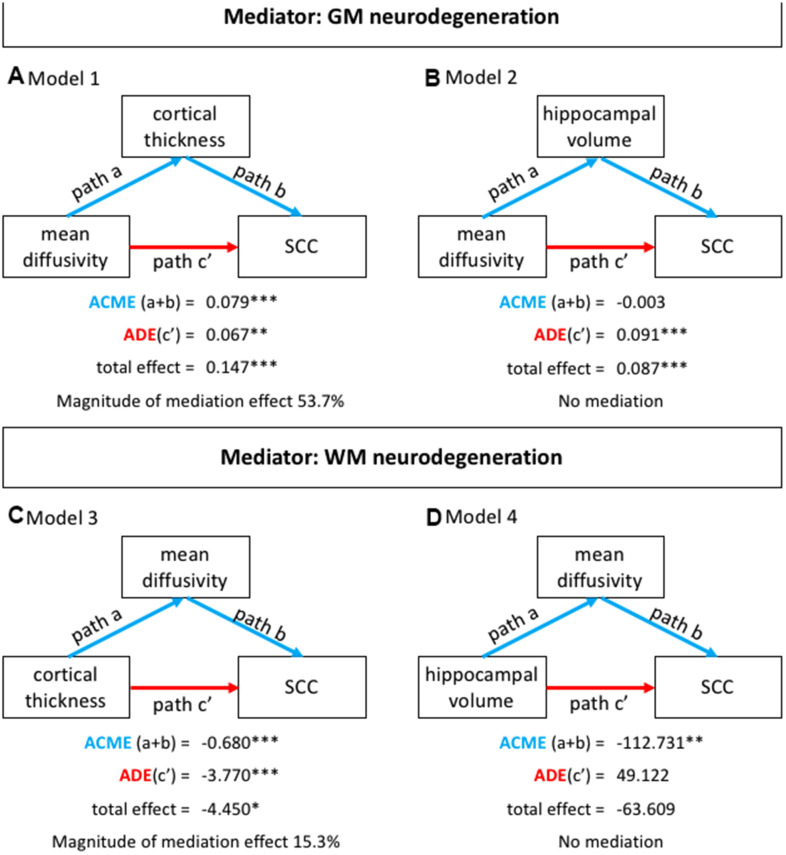
**Mediation analysis.** (**A**) Represents mediation model 1: subjective cognitive complaints as the dependent variable (Y), the average MD as the independent variable (X), and the average cortical thickness as the mediator (M); (**B**) Represents mediation model 2: subjective cognitive complaints as the dependent variable (Y), the average MD as the independent variable (X), and the TIV-corrected hippocampal volume (left+right) as the mediator (M). (**C**) Represents mediation model 3: subjective cognitive complaints as the dependent variable (Y), cortical thickness as the independent variable (X), and the average MD as the mediator (M); (**D**) Represents mediation model 4: subjective cognitive complaints as the dependent variable (Y), the TIV-corrected hippocampal volume (left+right) as the independent variable (X), and the average MD as the mediator (M). Note: age was not a significant covariate in models 1 and 3. SCC: subjective cognitive complaints; ACME: average causal mediation effect; ADE: average direct effect; M: mediator; Magnitude of the mediation effect: ACME / total effect; X: independent variable; Y: dependent variable. *p<0.05; **p<0.01; ***p<0.001.

The average MD was negatively correlated with the average cortical thickness (condition 1 of mediation analysis, Figure 2A, 2C and Table 3), indicating that a higher MD was associated with thinner cortex. The average MD was also negatively correlated with the hippocampal volume (condition 1 of mediation analysis, Figure 2B, 2D and Table 3), indicating that a higher MD was associated with a smaller hippocampal volume. Consistent with the voxel-based TBSS analysis, the test for condition 2 of the mediation analysis (Figure 2A, 2B) showed that the average MD was positively correlated with complaints, indicating that a higher MD was associated with a higher number of subjective cognitive complaints (Table 3). Likewise, the average cortical thickness and hippocampal volume were both negatively correlated with the number of complaints, indicating that cortical thinning and reduced hippocampal volume were associated with a higher number of subjective cognitive complaints (condition 2 of mediation analysis, Figure 2C, 2D and Table 3).

Mediation analysis showed that the average cortical thickness partially mediated the association between the average MD and the number of subjective cognitive complaints (mediation model 1, Figure 2A). Age was not a significant covariate in this model. Further, hippocampal volume was not a significant mediator of the association between the average MD and the number of subjective cognitive complaints (model 2, Figure 2B). The average MD partially mediated the association between the average cortical thickness and the number subjective cognitive complaints (model 3, Figure 2C). Age was not a significant covariate in this model. Finally, the total effect in model 4 was not significant (Figure 2D), meaning that this model was completely driven by the association between average MD and the number of subjective cognitive complaints.

The multiple linear regression model was significant (F_(1, 222)_=39.7; p<0.001, R^2^=0.263). Congruent with the mediation analyses, the number of complaints was mainly predicted by the average cortical thickness (ß= -0.417; p<0.001) and the average MD (ß= 0.163; p=0.01). Hippocampal volume (ß= 0.063; p=0.322) and age (ß= 0.058; p=0.459) were not significant predictors of the number of complaints in this multiple linear regression model.

## DISCUSSION

We investigated the interplay between GM and WM neurodegeneration in SCD, including thickness across the whole cortical mantle, hippocampal volume, and integrity across the whole WM skeleton. We found that the association of WM neurodegeneration with a higher number of complaints was widespread across the WM skeleton, with a tendency to spare the occipital lobe. In contrast, the negative association between GM neurodegeneration and the number of complaints was limited to frontal areas, the insula, and some temporal areas, including the hippocampus. Our analyses showed that GM and WM neurodegeneration were negatively associated with each other and both contributed similarly to the number of complaints, although the contribution of GM neurodegeneration (cortical thickness) was stronger as illustrated by a greater mediation effect and a higher beta value in the regression analysis.

In the current study, a higher number of subjective cognitive complaints was associated with cortical thinning in bilateral frontal and right lateral superior temporal areas, as well as in the insula. We also found a negative association between the number of complaints and hippocampal volume. However, there were no associations with other areas typically involved in AD, such as the entorhinal cortex and inferior parietal gyrus. This contrasts with previous studies on SCD, which reported a significant association between complaints and cortical thinning in the inferior parietal, inferior temporal, and middle temporal areas [14, 15]. This discrepancy could be explained by the fact that most of the previous SCD studies had a strong focus on AD: they included patients from memory clinics, operationalized SCD mostly based on episodic memory complaints, and constrained their analysis to brain areas typically affected in AD [7, 14–18, 37–41]. In contrast, our cohort is community-based, we operationalized SCD through complaints in cognitive domains beyond episodic memory, and analyzed the whole cortical mantle. In concordance with our results, a previous study analyzing the whole cortical mantle showed that SCD individuals had a widespread pattern of cortical thinning involving frontal, temporal, and parietal areas [22]. Altogether, these findings highlight that SCD is a heterogeneous entity where cortical thinning might be determined by multiple factors. One of the most prominent determinants of subjective cognitive complaints in community-based samples is older age [9, 42]. Our findings showed that older age was associated with GM neurodegeneration in most of the cortex, including most of the areas that were associated with the number of complaints. Interestingly, the only area associated with the number of complaints that was not associated with age was the right lateral temporal area. Recently, Lim et al. [28] showed that, the only structural difference between SCD individuals who progressed to MCI/dementia over 5 years and those who remained stable was cortical thinning in right lateral temporal areas. These findings highlight the need to take multiple cortical areas into consideration to gain a better understanding of neurobiological processes underlying SCD in heterogeneous populations.

In addition, we found that the number of subjective cognitive complaints was associated with worse WM integrity in widespread areas, clearly exceeding the areas associated with GM neurodegeneration. In particular, we observed that a higher number of cognitive complaints was associated with increased MD in most of the WM skeleton, with a tendency to spare the occipital lobe. A recent study also reported widespread WM degeneration in SCD [22]. Similar to the findings for GM neurodegeneration, we found that age was positively associated with WM neurodegeneration. Interestingly, the association between the number of subjective cognitive complaints and WM neurodegeneration exceeded the effect of age in posterior brain areas and internal capsule, while age was primarily associated with WM neurodegeneration in anterior brain areas, in our study. Age-related WM neurodegeneration has been primarily associated to changes in anterior WM tracts [43]. On the contrary, posterior WM tracts and the internal capsule are relatively spared in normal aging [36, 43], but they are prone to brain pathologies such as cerebrovascular disease and/or cerebral amyloid angiopathy (CAA) [44, 45]. Further, patients with dementia with Lewy bodies have worse WM integrity in posterior tracts like the ones identified in our current study [46]. In addition, AD patients with prominent cortical atrophy (i.e., the hippocampal-sparing subtype of AD) are prone to have WM lesions in posterior brain areas [47]. The internal capsule is an area especially vulnerable to microvascular damage [48]. Both the internal capsule and posterior WM tracts receive dense cholinergic input, and increased MD in cholinergic WM pathways was associated with greater cerebrovascular disease and lower cognitive performance in our cohort [49]. Finally, as we showed in a recent study using the same cohort, the association between the number of subjective cognitive complaints and WM neurodegeneration was independent from depressive symptomatology [50], despite depressive symptomatology is consistently associated with SCD [9, 12, 13]. All together, these findings indicate that SCD is associated with WM neurodegeneration in certain regions that go beyond age- or depressive symptomatology-related effects.

Another novel contribution of the current study is the analysis on the interplay between GM neurodegeneration and WM neurodegeneration. To our knowledge, only one previous study has investigated the association between GM neurodegeneration and WM neurodegeneration in SCD [23]. Hong et al. (2016) included a small cohort of 46 SCD patients with episodic memory complaints, of whom 41% had a high risk of progressing to AD based on age, *APOE* genotype, and cognitive performance. They assessed neurodegeneration in WM areas adjacent to the cortex using a ROI-based approach on DTI data. The authors reported that SCD patients at a high risk of progressing to AD had greater neurodegeneration in WM areas adjacent to frontotemporal and supramarginal cortices, and they did not find any differences in GM neurodegeneration [23]. Further, they demonstrated associations between several WM ROIs and an estimation of the average thickness across the whole cortical mantle. Our current study extends that approach by including a more fine-grained analysis of WM neurodegeneration at the voxel level, and we studied the association between GM neurodegeneration and WM neurodegeneration in areas exclusively associated with subjective cognitive complaints. We also extended the approach based on partial correlations in Hong et al. (2016) by applying mediation and multiple linear regression models, which provide richer information on inter-relationships among variables. We found that cortical thickness and MD were negatively associated with each other and jointly contributed to the number of subjective cognitive complaints. However, the magnitude of the mediation and the beta value of cortical thickness were the highest, suggesting that GM neurodegeneration has a stronger contribution to SCD as compared with the contribution of WM neurodegeneration. In prodromal AD, GM neurodegeneration seems to be downstream to WM neurodegeneration in longitudinal studies [51]. In cross-sectional studies, this finding may be reflected by a stronger association between GM neurodegeneration and cognition, and a weaker association between WM neurodegeneration and cognition [52]. Hence, our current findings could be interpreted as WM neurodegeneration preceding GM neurodegeneration during the stage of SCD. Further, we observed that WM neurodegeneration was widespread across the WM skeleton and GM neurodegeneration was limited to frontotemporal areas. Despite our WM maker is a microstructural measure and our GM marker is a macrostructural measure, this finding could also suggest a more advanced neurodegenerative process in the WM than in the GM. Altogether, these data suggest that WM neurodegeneration might start earlier than GM neurodegeneration, and SCD seems to be a sensitive behavioral marker of heterogeneous processes of neurodegeneration.

Some limitations should be noted. Although we report novel data on the interplay between GM neurodegeneration and WM neurodegeneration in SCD, the interpretation of WM neurodegeneration possibly preceding GM neurodegeneration needs to be confirmed in longitudinal studies. Follow-up data is being collected in the GENIC cohort to address this question in the future. Our current analyses showed an association of the number of complaints with GM and WM neurodegeneration in brain areas other than those typically targeted by AD, which may suggest the contribution of non-AD pathologies. Because multiple pathologies usually coexist in the brain of cognitively unimpaired individuals [53, 54], future studies should unveil the pathologies underlying non-AD patterns of GM and WM neurodegeneration in SCD. Investigating pathologies such as cerebrovascular disease and tauopathies is warranted due to their contribution to SCD in community-based cohorts [11, 49, 50, 55]. A limitation of our cohort is that we do not have biomarkers for amyloid-beta and tau-related pathologies.

This study is one of the few in investigating the association between GM and WM neurodegeneration in SCD. Our data suggest an association between neurodegeneration processes affecting the GM and WM in SCD individuals. However, GM neurodegeneration seemed to have a stronger contribution to SCD in our community-based cohort, highlighting brain areas that are typically not targeted by AD. This finding suggests the contribution of non-AD pathologies to SCD, and encourages that future studies extend imaging analysis to brain areas other than those typically involved in AD.

## MATERIALS AND METHODS

### Participants

A total of 225 individuals were selected from the GENIC cohort [56], a community-based study from the Canary Islands (Spain). Inclusion criteria for the current study were in accord with the basic SCD criteria published by the SCD initiative (SCD-I) working group [4]: (1) Normal cognitive performance in comprehensive neuropsychological assessment using pertinent clinical normative data (i.e., individuals did not fulfill cognitive criteria for mild cognitive impairment or dementia); (2) preserved activities of daily living and global cognition, operationalized as a Blessed Rating Dementia Scale (BRDS) [57] score ≤4, a Functional Activity Questionnaire (FAQ) [58] score ≤5, and a Mini-Mental State Examination (MMSE) [59] score ≥24; (3) No abnormal findings such as stroke, tumors, hippocampal sclerosis, etc., in MRI according to an experienced neuroradiologist; (4) no medical history of neurological or psychiatric disorders (including a diagnosis of major depression), systemic diseases or head trauma; and (5) no history of substance abuse. We also required all participants to have MRI data available, including three-dimensional T1-weigthed and diffusion tensor imaging (DTI) sequences (please see below). Participants with available MRI data in GENIC cohort tend to be younger and more educated compared to those without MRI [9]. Participants’ recruitment in the GENIC cohort was done through primary care health centers, advertisements in local schools, and relatives and acquaintances of the research staff, covering a representative sample in terms of age, sex, and education. Participation was completely voluntary and all the participants gave written informed consent approved by the local ethics committee.

### Subjective cognitive complaints

Subjective cognitive complaints were assessed through a questionnaire covering complaints about memory, orientation, executive functions, face recognition, language production, language comprehension, word-finding, reading and writing (Table 4) [9, 60]. All participants answered nine yes/no questions referred to cognitive changes that occurred approximately during the last six months. Each answer was coded as 0 (absence of complaint) or 1 (presence of complaint. Answers were summed up and the total number of complaints was obtained and used as input for the statistical analysis. Further details regarding the distribution of subjective cognitive complaints across the different cognitive domains has previously been described elsewhere [60].

**Table 4:** Questions to assess subjective cognitive complaints in the GENIC cohort.

Orientation	1-. Do you find it harder to orient yourself in time or space?
Memory	2-. Do you have memory problems?
Visuoperception	3-. Do you find it harder to recognize familiar faces or people you do not see often?
Executive Functions	4-. Do you find it harder to manage money or do mental arithmetic?
Language	5-. Do you find it harder to find words?
	6-. Do you have any problems with reading?
	7-. Do you have any problems with writing?
	8-. Have you noticed whether you speak less or worse lately?
	9-. Do you find it harder to follow a conversation? Do you find it harder to understand what people say to you?

In this study, the term SCD is used when referring to the clinical entity or concept of SCD; and the term subjective cognitive complaints is used when referring to the variable used in our statistical analyses. The continuous variable of subjective cognitive complaints was preferred to the dichotomous variable of SCD due to the nature of our statistical models and to avoid arbitrary clinical thresholds.

### Magnetic resonance imaging (MRI)

Participants were scanned using a 3.0T GE imaging system (General Electric, Milwaukee, WI, USA) located at the *Hospital Universitario de Canarias* in Tenerife, Spain. A three-dimensional T1-weighted Fast Spoiled Gradient Echo (FSPGR) sequence and a DTI sequence were acquired in sagittal and axial planes, respectively. The parameters were as follows, T1-weighted: repetition time/echo time/inversion time = 8.73/1.74/650 ms., field of view = 250 x 250 mm, matrix = 250 x 250 mm, flip angle = 12°, slice thickness = 1 mm; DTI: repetition time/echo time = 15000/≈72 ms., field of view = 256 × 256 mm, matrix = 128 × 128 mm, 31 directions, B value = 1000, flip angle = 90°, slice thickness = 2.4 mm. Full brain and skull coverage was required for the MRI datasets and detailed quality control was carried out on all the images according to previously published criteria [61].

The T1-weighted images were processed and analyzed with the FreeSurfer 6.0.0 image analysis suite (http://surfer.nmr.mgh.harvard.edu/). The hippocampal volume (left+right) was selected for this study, divided by the estimated total intracranial volume (TIV) to account for variability in head size [62]. Statistical analyses were also performed across the cortical mantle. DTI data were processed and analyzed with the FSL software (https://fsl.fmrib.ox.ac.uk/fsl/fslwiki/), using the FDT and TBSS tools. The measure of MD was selected for statistical analysis. MD is an early indicator of neurodegeneration and is more sensitive to changes during preclinical AD and SCD stages as compared with other diffusivity measures, including fractional anisotropy [63, 64]. Furthermore, the MD index has previously demonstrated an association with cognitive performance in the GENIC cohort [49, 65]. Careful visual quality control was performed on all the output data obtained from FreeSurfer and FSL, and manual edits were done when appropriate. TheHiveDB was used for data management and processing in this study [66].

### Statistical analysis

To address the aim of investigating the association between subjective cognitive complaints and cortical thickness across the whole cortical mantle, a vertex-wise analysis was performed using the FreeSurfer software. We also conducted a separate vertex-wise analysis for the age variable and compared the overlap between the cortical maps obtained for subjective cognitive complaints and age. A general linear model was fitted at each vertex using cortical thickness as the dependent variable and subjective cognitive complaints or age as the independent variables. Permutations-based non-parametric tests with 5000 iterations were used with a cluster-forming threshold of p≤0.01 (two-sided) using the family wise error (FWE) correction for multiple comparisons (p≤0.05). The smoothing kernel (full width at half maximum, FWHM) was equal to 10 mm. Cortical thickness values of statistically significant clusters associated with subjective complaints were transformed into individuals’ native space for computation of within-clusters average thickness used in subsequent analyses (from here, referred to as ‘average cortical thickness’).

To address the aim of investigating the association between subjective cognitive complaints and hippocampal volume (TIV corrected), we computed the Pearson correlation between the two variables. We also computed the Pearson correlation between hippocampal volume (TIV corrected) and the age variable.

To address the aim of investigating the association between subjective cognitive complaints and integrity across the whole WM skeleton, a voxel-based analysis on the white matter skeleton was performed using the FSL software. We also conducted a separate voxel-based analysis for the age variable and compared the overlap between the skeleton maps obtained for subjective cognitive complaints and age. A general linear model was fitted at each voxel using MD as the dependent variable and subjective cognitive complaints or age as the independent variables. Permutation-based non-parametric testing with 5000 iterations was used followed by threshold-free cluster enhancement (TFCE) and the family-wise error (FWE) correction for multiple comparisons (p≤0.01, two-sided). MD values of statistically significant clusters associated with subjective complaints in individual’s native space were used to compute within-clusters average MD values for subsequent analyses (from here, referred to as ‘average MD’).

To address the aim of investigating the interplay between GM neurodegeneration and WM neurodegeneration, we developed an approach based on mediation models and multiple linear regression as described below.

The only previous study investigating the association between GM neurodegeneration and WM neurodegeneration in SCD used partial correlation analyses [23]. However, correlation analyses are limited when it comes to fully understand the way in how GM and WM neurodegeneration have an effect on each other and their joint contribution towards subjective cognitive complaints. A strength of our study is that we extended that approach by using mediation analysis. The advantage of mediation analysis is the possibility to ascertain the unique and combined contribution of GM and WM neurodegeneration towards complaints. Further, by testing complementary models it can be studied whether one of the neurodegeneration markers is the main driver of the contribution towards complaints. We specifically tested: (i) whether GM neurodegeneration mediates the association between WM neurodegeneration and subjective cognitive complaints; and (ii) whether WM neurodegeneration mediates the association between GM neurodegeneration and subjective cognitive complaints. Mediation analysis were conducted using the “Mediation” R package [67]. The TIV-corrected hippocampal volume (left+right), and the average cortical thickness and average MD of statistically significant clusters (see above) were used as the input data for mediation analysis. All mediation models are represented in Figure 1. In order to investigate the role of age in our analyses, the four mediation models were fitted with and without age as a covariate.

The three basic conditions of mediation analysis were tested with simple and multiple linear regression models [68]. Mediation were interpreted was based on the average direct effect (ADE), the average causal mediation effect (ACME), and the total effect. Briefly, the ADE represents the direct effect of the independent variable on subjective cognitive complaints, while the ACME represents the indirect effect of the independent variable on subjective cognitive complaints, through the mediator variable. The total effect represents the sum of the ACME and the ADE. When the ACME is statistically significant (in conjunction with a significant total effect) there is a mediation effect that can be of two types: full mediation, when the ACME is significant but the ADE is not significant; and partial mediation, when both the ACME and the ADE are significant [67]. The ACME and the ADE were calculated by using confidence intervals based on non-parametric bootstrap sampling (1000 simulations). We also calculated the magnitude of the mediation effect by dividing ACME by the total effect (Figure 2).

In addition, we applied multiple linear regression to investigate the partial association of the average cortical thickness, hippocampal volume, the average MD, and age with subjective cognitive complaints. We used the backwards option with the best general lineal model - *bestglm* - method for variables exit.

A p-value ≤0.05 (two-tailed) was considered significant in all these analyses.

### Ethical approval

Participation was completely voluntary, and all the participants gave written informed consent approved by the local ethics committee.

## References

[1] Villemagne VL, Burnham S, Bourgeat P, Brown B, Ellis KA, Salvado O, Szoeke C, Macaulay SL, Martins R, Maruff P, Ames D, Rowe CC, Masters CL, and Australian Imaging Biomarkers and Lifestyle (AIBL) Research Group. Amyloid β deposition, neurodegeneration, and cognitive decline in sporadic Alzheimer's disease: a prospective cohort study. Lancet Neurol. 2013; 12:357–67. 10.1016/S1474-4422(13)70044-9 23477989

[2] Reisberg B, Shulman MB, Torossian C, Leng L, Zhu W. Outcome over seven years of healthy adults with and without subjective cognitive impairment. Alzheimers Dement. 2010; 6:11–24. 10.1016/j.jalz.2009.10.002 20129317 PMC3873197

[3] Donovan NJ, Amariglio RE, Zoller AS, Rudel RK, Gomez-Isla T, Blacker D, Hyman BT, Locascio JJ, Johnson KA, Sperling RA, Marshall GA, Rentz DM. Subjective cognitive concerns and neuropsychiatric predictors of progression to the early clinical stages of Alzheimer disease. Am J Geriatr Psychiatry. 2014; 22:1642–51. 10.1016/j.jagp.2014.02.007 24698445 PMC4145054

[4] Jessen F, Amariglio RE, van Boxtel M, Breteler M, Ceccaldi M, Chételat G, Dubois B, Dufouil C, Ellis KA, van der Flier WM, Glodzik L, van Harten AC, de Leon MJ, et al, and Subjective Cognitive Decline Initiative (SCD-I) Working Group. A conceptual framework for research on subjective cognitive decline in preclinical Alzheimer’s disease. Alzheimers Dement. 2014; 10:844–52. 10.1016/j.jalz.2014.01.001 24798886 PMC4317324

[5] Perrotin A, La Joie R, de La Sayette V, Barré L, Mézenge F, Mutlu J, Guilloteau D, Egret S, Eustache F, Chételat G. Subjective cognitive decline in cognitively normal elders from the community or from a memory clinic: Differential affective and imaging correlates. Alzheimers Dement. 2017; 13:550–60. 10.1016/j.jalz.2016.08.011 27693187

[6] Amariglio RE, Mormino EC, Pietras AC, Marshall GA, Vannini P, Johnson KA, Sperling RA, Rentz DM. Subjective cognitive concerns, amyloid-β, and neurodegeneration in clinically normal elderly. Neurology. 2015; 85:56–62. 10.1212/WNL.0000000000001712 26048028 PMC4501939

[7] Snitz BE, Lopez OL, McDade E, Becker JT, Cohen AD, Price JC, Mathis CA, Klunk WE. Amyloid-β Imaging in Older Adults Presenting to a Memory Clinic with Subjective Cognitive Decline: A Pilot Study. J Alzheimers Dis. 2015 (Suppl 1); 48:S151–59. 10.3233/JAD-150113 26402082 PMC4675050

[8] Benedictus MR, van Harten AC, Leeuwis AE, Koene T, Scheltens P, Barkhof F, Prins ND, van der Flier WM. White Matter Hyperintensities Relate to Clinical Progression in Subjective Cognitive Decline. Stroke. 2015; 46:2661–64. 10.1161/STROKEAHA.115.009475 26173729

[9] Cedres N, Machado A, Molina Y, Diaz-Galvan P, Hernández-Cabrera JA, Barroso J, Westman E, Ferreira D. Subjective Cognitive Decline Below and Above the Age of 60: A Multivariate Study on Neuroimaging, Cognitive, Clinical, and Demographic Measures. J Alzheimers Dis. 2019; 68:295–309. 10.3233/JAD-180720 30741680

[10] Diniz BS, Butters MA, Albert SM, Dew MA, Reynolds CF 3rd. Late-life depression and risk of vascular dementia and Alzheimer’s disease: systematic review and meta-analysis of community-based cohort studies. Br J Psychiatry. 2013; 202:329–35. 10.1192/bjp.bp.112.118307 23637108 PMC3640214

[11] Buckley RF, Hanseeuw B, Schultz AP, Vannini P, Aghjayan SL, Properzi MJ, Jackson JD, Mormino EC, Rentz DM, Sperling RA, Johnson KA, Amariglio RE. Region-Specific Association of Subjective Cognitive Decline With Tauopathy Independent of Global β-Amyloid Burden. JAMA Neurol. 2017; 74:1455–63. 10.1001/jamaneurol.2017.2216 28973551 PMC5774633

[12] Ginó S, Mendes T, Maroco J, Ribeiro F, Schmand BA, de Mendonça A, Guerreiro M. Memory complaints are frequent but qualitatively different in young and elderly healthy people. Gerontology. 2010; 56:272–77. 10.1159/000240048 19776545

[13] Zlatar ZZ, Moore RC, Palmer BW, Thompson WK, Jeste DV. Cognitive complaints correlate with depression rather than concurrent objective cognitive impairment in the successful aging evaluation baseline sample. J Geriatr Psychiatry Neurol. 2014; 27:181–87. 10.1177/0891988714524628 24614203 PMC4255945

[14] Saykin AJ, Wishart HA, Rabin LA, Santulli RB, Flashman LA, West JD, McHugh TL, Mamourian AC. Older adults with cognitive complaints show brain atrophy similar to that of amnestic MCI. Neurology. 2006; 67:834–42. 10.1212/01.wnl.0000234032.77541.a2 16966547 PMC3488276

[15] Stewart R, Godin O, Crivello F, Maillard P, Mazoyer B, Tzourio C, Dufouil C. Longitudinal neuroimaging correlates of subjective memory impairment: 4-year prospective community study. Br J Psychiatry. 2011; 198:199–205. 10.1192/bjp.bp.110.078683 21357878

[16] van der Flier WM, van Buchem MA, Weverling-Rijnsburger AW, Mutsaers ER, Bollen EL, Admiraal-Behloul F, Westendorp RG, Middelkoop HA. Memory complaints in patients with normal cognition are associated with smaller hippocampal volumes. J Neurol. 2004; 251:671–75. 10.1007/s00415-004-0390-7 15311341

[17] Meiberth D, Scheef L, Wolfsgruber S, Boecker H, Block W, Träber F, Erk S, Heneka MT, Jacobi H, Spottke A, Walter H, Wagner M, Hu X, Jessen F. Cortical thinning in individuals with subjective memory impairment. J Alzheimers Dis. 2015; 45:139–46. 10.3233/JAD-142322 25471190

[18] Schultz SA, Oh JM, Koscik RL, Dowling NM, Gallagher CL, Carlsson CM, Bendlin BB, LaRue A, Hermann BP, Rowley HA, Asthana S, Sager MA, Johnson SC, Okonkwo OC. Subjective memory complaints, cortical thinning, and cognitive dysfunction in middle-aged adults at risk for AD. Alzheimers Dement (Amst). 2015; 1:33–40. 10.1016/j.dadm.2014.11.010 25938132 PMC4412027

[19] Verfaillie SC, Slot RE, Tijms BM, Bouwman F, Benedictus MR, Overbeek JM, Koene T, Vrenken H, Scheltens P, Barkhof F, van der Flier WM. Thinner cortex in patients with subjective cognitive decline is associated with steeper decline of memory. Neurobiol Aging. 2018; 61:238–44. 10.1016/j.neurobiolaging.2017.09.009 29029762

[20] Ferreira D, Falahati F, Linden C, Buckley RF, Ellis KA, Savage G, Villemagne VL, Rowe CC, Ames D, Simmons A, Westman E. A 'Disease Severity Index' to identify individuals with Subjective Memory Decline who will progress to mild cognitive impairment or dementia. Sci Rep. 2017; 7:44368. 10.1038/srep44368 28287184 PMC5347012

[21] Peter J, Scheef L, Abdulkadir A, Boecker H, Heneka M, Wagner M, Koppara A, Klöppel S, Jessen F, and Alzheimer’s Disease Neuroimaging Initiative. Gray matter atrophy pattern in elderly with subjective memory impairment. Alzheimers Dement. 2014; 10:99–108. 10.1016/j.jalz.2013.05.1764 23867795

[22] Hong YJ, Yoon B, Shim YS, Ahn KJ, Yang DW, Lee JH. Gray and White Matter Degenerations in Subjective Memory Impairment: Comparisons with Normal Controls and Mild Cognitive Impairment. J Korean Med Sci. 2015; 30:1652–58. 10.3346/jkms.2015.30.11.1652 26539011 PMC4630483

[23] Hong YJ, Kim CM, Jang EH, Hwang J, Roh JH, Lee JH. White Matter Changes May Precede Gray Matter Loss in Elderly with Subjective Memory Impairment. Dement Geriatr Cogn Disord. 2016; 42:227–35. 10.1159/000450749 27701163

[24] Kiuchi K, Kitamura S, Taoka T, Yasuno F, Tanimura M, Matsuoka K, Ikawa D, Toritsuka M, Hashimoto K, Makinodan M, Kosaka J, Morikawa M, Kichikawa K, Kishimoto T. Gray and white matter changes in subjective cognitive impairment, amnestic mild cognitive impairment and Alzheimer’s disease: a voxel-based analysis study. PLoS One. 2014; 9:e104007. 10.1371/journal.pone.0104007 25093415 PMC4122459

[25] Sun Y, Dai Z, Li Y, Sheng C, Li H, Wang X, Chen X, He Y, Han Y. Subjective Cognitive Decline: Mapping Functional and Structural Brain Changes-A Combined Resting-State Functional and Structural MR Imaging Study. Radiology. 2016; 281:185–92. 10.1148/radiol.2016151771 27002419

[26] Valech N, Sánchez-Benavides G, Tort-Merino A, Coll-Padrós N, Olives J, León M, Falcon C, Molinuevo JL, Rami L. Associations Between the Subjective Cognitive Decline-Questionnaire’s Scores, Gray Matter Volume, and Amyloid-β Levels. J Alzheimers Dis. 2019; 72:1287–302. 10.3233/JAD-190624 31707366

[27] Sánchez-Benavides G, Grau-Rivera O, Suárez-Calvet M, Minguillon C, Cacciaglia R, Gramunt N, Falcon C, Gispert JD, Molinuevo JL, and ALFA Study. Brain and cognitive correlates of subjective cognitive decline-plus features in a population-based cohort. Alzheimers Res Ther. 2018; 10:123. 10.1186/s13195-018-0449-9 30572953 PMC6302483

[28] Lim EY, Shim YS, Hong YJ, Ryu SY, Cho AH, Yang DW. Different Cortical Thinning Patterns Depending on Their Prognosis in Individuals with Subjective Cognitive Decline. Dement Neurocogn Disord. 2019; 18:113–21. 10.12779/dnd.2019.18.4.113 31942170 PMC6946618

[29] Jung NY, Seo SW, Yoo H, Yang JJ, Park S, Kim YJ, Lee J, Lee JS, Jang YK, Lee JM, Kim ST, Kim S, Kim EJ, et al. Classifying anatomical subtypes of subjective memory impairment. Neurobiol Aging. 2016; 48:53–60. 10.1016/j.neurobiolaging.2016.08.010 27639121

[30] Fan LY, Lai YM, Chen TF, Hsu YC, Chen PY, Huang KZ, Cheng TW, Tseng WI, Hua MS, Chen YF, Chiu MJ. Diminution of context association memory structure in subjects with subjective cognitive decline. Hum Brain Mapp. 2018; 39:2549–62. 10.1002/hbm.24022 29516634 PMC6866359

[31] Li XY, Tang ZC, Sun Y, Tian J, Liu ZY, Han Y. White matter degeneration in subjective cognitive decline: a diffusion tensor imaging study. Oncotarget. 2016; 7:54405–14. 10.18632/oncotarget.10091 27384675 PMC5342351

[32] Brueggen K, Dyrba M, Cardenas-Blanco A, Schneider A, Fliessbach K, Buerger K, Janowitz D, Peters O, Menne F, Priller J, Spruth E, Wiltfang J, Vukovich R, et al, and DELCODE Study Group. Structural integrity in subjective cognitive decline, mild cognitive impairment and Alzheimer's disease based on multicenter diffusion tensor imaging. J Neurol. 2019; 266:2465–74. 10.1007/s00415-019-09429-3 31227891

[33] Ryu SY, Lim EY, Na S, Shim YS, Cho JH, Yoon B, Hong YJ, Yang DW. Hippocampal and entorhinal structures in subjective memory impairment: a combined MRI volumetric and DTI study. Int Psychogeriatr. 2017; 29:785–92. 10.1017/S1041610216002349 28067183

[34] Wang XN, Zeng Y, Chen GQ, Zhang YH, Li XY, Hao XY, Yu Y, Zhang M, Sheng C, Li YX, Sun Y, Li HY, Song Y, et al. Abnormal organization of white matter networks in patients with subjective cognitive decline and mild cognitive impairment. Oncotarget. 2016; 7:48953–62. 10.18632/oncotarget.10601 27418146 PMC5226483

[35] Salat DH, Buckner RL, Snyder AZ, Greve DN, Desikan RS, Busa E, Morris JC, Dale AM, Fischl B. Thinning of the cerebral cortex in aging. Cereb Cortex. 2004; 14:721–30. 10.1093/cercor/bhh032 15054051

[36] Salat DH, Tuch DS, Greve DN, van der Kouwe AJ, Hevelone ND, Zaleta AK, Rosen BR, Fischl B, Corkin S, Rosas HD, Dale AM. Age-related alterations in white matter microstructure measured by diffusion tensor imaging. Neurobiol Aging. 2005; 26:1215–27. 10.1016/j.neurobiolaging.2004.09.017 15917106

[37] Verfaillie SC, Tijms B, Versteeg A, Benedictus MR, Bouwman FH, Scheltens P, Barkhof F, Vrenken H, van der Flier WM. Thinner temporal and parietal cortex is related to incident clinical progression to dementia in patients with subjective cognitive decline. Alzheimers Dement (Amst). 2016; 5:43–52. 10.1016/j.dadm.2016.10.007 28054027 PMC5198882

[38] Perrotin A, de Flores R, Lamberton F, Poisnel G, La Joie R, de la Sayette V, Mézenge F, Tomadesso C, Landeau B, Desgranges B, Chételat G. Hippocampal Subfield Volumetry and 3D Surface Mapping in Subjective Cognitive Decline. J Alzheimers Dis. 2015 (Suppl 1); 48:S141–50. 10.3233/JAD-150087 26402076

[39] Jessen F, Feyen L, Freymann K, Tepest R, Maier W, Heun R, Schild HH, Scheef L. Volume reduction of the entorhinal cortex in subjective memory impairment. Neurobiol Aging. 2006; 27:1751–56. 10.1016/j.neurobiolaging.2005.10.010 16309795

[40] Selnes P, Aarsland D, Bjørnerud A, Gjerstad L, Wallin A, Hessen E, Reinvang I, Grambaite R, Auning E, Kjærvik VK, Due-Tønnessen P, Stenset V, Fladby T. Diffusion tensor imaging surpasses cerebrospinal fluid as predictor of cognitive decline and medial temporal lobe atrophy in subjective cognitive impairment and mild cognitive impairment. J Alzheimers Dis. 2013; 33:723–36. 10.3233/JAD-2012-121603 23186987

[41] Rabin LA, Smart CM, Crane PK, Amariglio RE, Berman LM, Boada M, Buckley RF, Chételat G, Dubois B, Ellis KA, Gifford KA, Jefferson AL, Jessen F, et al. Subjective Cognitive Decline in Older Adults: An Overview of Self-Report Measures Used Across 19 International Research Studies. J Alzheimers Dis. 2015 (Suppl 1); 48:S63–86. 10.3233/JAD-150154 26402085 PMC4617342

[42] Zwan MD, Villemagne VL, Doré V, Buckley R, Bourgeat P, Veljanoski R, Salvado O, Williams R, Margison L, Rembach A, Macaulay SL, Martins R, Ames D, et al. Subjective Memory Complaints in APOEɛ4 Carriers are Associated with High Amyloid-β Burden. J Alzheimers Dis. 2016; 49:1115–22. 10.3233/JAD-150446 26639956

[43] Hoagey DA, Rieck JR, Rodrigue KM, Kennedy KM. Joint contributions of cortical morphometry and white matter microstructure in healthy brain aging: A partial least squares correlation analysis. Hum Brain Mapp. 2019; 40:5315–29. 10.1002/hbm.24774 31452304 PMC6864896

[44] Caplan LR. Lacunar infarction and small vessel disease: pathology and pathophysiology. J Stroke. 2015; 17:2–6. 10.5853/jos.2015.17.1.2 25692102 PMC4325635

[45] Thanprasertsuk S, Martinez-Ramirez S, Pontes-Neto OM, Ni J, Ayres A, Reed A, Swords K, Gurol ME, Greenberg SM, Viswanathan A. Posterior white matter disease distribution as a predictor of amyloid angiopathy. Neurology. 2014; 83:794–800. 10.1212/WNL.0000000000000732 25063759 PMC4155043

[46] Watson R, Blamire AM, Colloby SJ, Wood JS, Barber R, He J, O’Brien JT. Characterizing dementia with Lewy bodies by means of diffusion tensor imaging. Neurology. 2012; 79:906–14. 10.1212/WNL.0b013e318266fc51 22895591 PMC3425843

[47] Ferreira D, Shams S, Cavallin L, Viitanen M, Martola J, Granberg T, Shams M, Aspelin P, Kristoffersen-Wiberg M, Nordberg A, Wahlund LO, Westman E. The contribution of small vessel disease to subtypes of Alzheimer’s disease: a study on cerebrospinal fluid and imaging biomarkers. Neurobiol Aging. 2018; 70:18–29. 10.1016/j.neurobiolaging.2018.05.028 29935417

[48] Badji A, Sabra D, Bherer L, Cohen-Adad J, Girouard H, Gauthier CJ. Arterial stiffness and brain integrity: A review of MRI findings. Ageing Res Rev. 2019; 53:100907. 10.1016/j.arr.2019.05.001 31063866

[49] Nemy M, Cedres N, Grothe MJ, Muehlboeck JS, Lindberg O, Nedelska Z, Stepankova O, Vyslouzilova L, Eriksdotter M, Barroso J, Teipel S, Westman E, Ferreira D. Cholinergic white matter pathways make a stronger contribution to attention and memory in normal aging than cerebrovascular health and nucleus basalis of Meynert. Neuroimage. 2020; 211:116607. 10.1016/j.neuroimage.2020.116607 32035186

[50] Diaz-Galvan P, Cedres N, Figueroa N, Barroso J, Westman E, Ferreira D. Cerebrovascular Disease and Depressive Symptomatology in Individuals With Subjective Cognitive Decline: A Community-Based Study. Front Aging Neurosci. 2021; 13:656990. 10.3389/fnagi.2021.656990 34385912 PMC8353130

[51] Archer DB, Moore EE, Shashikumar N, Dumitrescu L, Pechman KR, Landman BA, Gifford KA, Jefferson AL, Hohman TJ. Free-water metrics in medial temporal lobe white matter tract projections relate to longitudinal cognitive decline. Neurobiol Aging. 2020; 94:15–23. 10.1016/j.neurobiolaging.2020.05.001 32502831 PMC7483422

[52] Hong Z, Ng KK, Sim SK, Ngeow MY, Zheng H, Lo JC, Chee MW, Zhou J. Differential age-dependent associations of gray matter volume and white matter integrity with processing speed in healthy older adults. Neuroimage. 2015; 123:42–50. 10.1016/j.neuroimage.2015.08.034 26302672

[53] Boyle PA, Yang J, Yu L, Leurgans SE, Capuano AW, Schneider JA, Wilson RS, Bennett DA. Varied effects of age-related neuropathologies on the trajectory of late life cognitive decline. Brain. 2017; 140:804–12. 10.1093/brain/aww341 28082297 PMC5837473

[54] Schneider JA, Boyle PA, Arvanitakis Z, Bienias JL, Bennett DA. Subcortical infarcts, Alzheimer's disease pathology, and memory function in older persons. Ann Neurol. 2007; 62:59–66. 10.1002/ana.21142 17503514

[55] Jack CR Jr, Wiste HJ, Weigand SD, Therneau TM, Knopman DS, Lowe V, Vemuri P, Mielke MM, Roberts RO, Machulda MM, Senjem ML, Gunter JL, Rocca WA, Petersen RC. Age-specific and sex-specific prevalence of cerebral β-amyloidosis, tauopathy, and neurodegeneration in cognitively unimpaired individuals aged 50-95 years: a cross-sectional study. Lancet Neurol. 2017; 16:435–44. 10.1016/S1474-4422(17)30077-7 28456479 PMC5516534

[56] Gonzalez-Burgos L, Hernández-Cabrera JA, Westman E, Barroso J, Ferreira D. Cognitive compensatory mechanisms in normal aging: a study on verbal fluency and the contribution of other cognitive functions. Aging (Albany NY). 2019; 11:4090–106. 10.18632/aging.102040 31232698 PMC6628999

[57] Blessed G, Tomlinson BE, Roth M. The association between quantitative measures of dementia and of senile change in the cerebral grey matter of elderly subjects. Br J Psychiatry. 1968; 114:797–811. 10.1192/bjp.114.512.797 5662937

[58] Pfeffer RI, Kurosaki TT, Harrah CH Jr, Chance JM, Filos S. Measurement of functional activities in older adults in the community. J Gerontol. 1982; 37:323–29. 10.1093/geronj/37.3.323 7069156

[59] Folstein MF, Folstein SE, McHugh PR. “Mini-mental state”. A practical method for grading the cognitive state of patients for the clinician. J Psychiatr Res. 1975; 12:189–98. 10.1016/0022-3956(75)90026-6 1202204

[60] Diaz-Galvan P, Ferreira D, Cedres N, Falahati F, Hernández-Cabrera JA, Ames D, Barroso J, Westman E. Comparing different approaches for operationalizing subjective cognitive decline: impact on syndromic and biomarker profiles. Sci Rep. 2021; 11:4356. 10.1038/s41598-021-83428-1 33623075 PMC7902653

[61] Simmons A, Westman E, Muehlboeck S, Mecocci P, Vellas B, Tsolaki M, Kłoszewska I, Wahlund LO, Soininen H, Lovestone S, Evans A, Spenger C. The AddNeuroMed framework for multi-centre MRI assessment of Alzheimer’s disease: experience from the first 24 months. Int J Geriatr Psychiatry. 2011; 26:75–82. 10.1002/gps.2491 21157852

[62] Voevodskaya O, Simmons A, Nordenskjöld R, Kullberg J, Ahlström H, Lind L, Wahlund LO, Larsson EM, Westman E, and Alzheimer’s Disease Neuroimaging Initiative. The effects of intracranial volume adjustment approaches on multiple regional MRI volumes in healthy aging and Alzheimer’s disease. Front Aging Neurosci. 2014; 6:264. 10.3389/fnagi.2014.00264 25339897 PMC4188138

[63] Liu J, Yin C, Xia S, Jia L, Guo Y, Zhao Z, Li X, Han Y, Jia J. White matter changes in patients with amnestic mild cognitive impairment detected by diffusion tensor imaging. PLoS One. 2013; 8:e59440. 10.1371/journal.pone.0059440 23555673 PMC3605411

[64] Li X, Westman E, Ståhlbom AK, Thordardottir S, Almkvist O, Blennow K, Wahlund LO, Graff C. White matter changes in familial Alzheimer’s disease. J Intern Med. 2015; 278:211–18. 10.1111/joim.12352 25639959

[65] Ferreira D, Machado A, Molina Y, Nieto A, Correia R, Westman E, Barroso J. Cognitive Variability during Middle-Age: Possible Association with Neurodegeneration and Cognitive Reserve. Front Aging Neurosci. 2017; 9:188. 10.3389/fnagi.2017.00188 28649200 PMC5465264

[66] Muehlboeck JS, Westman E, Simmons A. TheHiveDB image data management and analysis framework. Front Neuroinform. 2014; 7:49. 10.3389/fninf.2013.00049 24432000 PMC3880907

[67] Tingley D, Yamamoto T, Hirose K, Keele L, Imai K. Mediation: R Package for Causal Mediation Analysis. J Stat Softw. 2014; 59:1-38. 10.18637/jss.v059.i05

[68] Baron RM, Kenny DA. The moderator-mediator variable distinction in social psychological research: conceptual, strategic, and statistical considerations. J Pers Soc Psychol. 1986; 51:1173–82. 10.1037//0022-3514.51.6.1173 3806354

